# Firearm Practices, Perceptions of Safety, and Opinions on Injury Prevention Strategies Among California Adults

**DOI:** 10.1001/jamanetworkopen.2021.19146

**Published:** 2021-08-03

**Authors:** Rocco Pallin, Garen J. Wintemute, Nicole Kravitz-Wirtz

**Affiliations:** 1 Violence Prevention Research Program, Department of Emergency Medicine, University of California Davis School of Medicine, Sacramento; 2 University of California Firearm Violence Research Center, Sacramento

## Abstract

**Importance:**

Safe firearm storage and other interventions may reduce pediatric firearm deaths and injuries.

**Objective:**

To compare firearm ownership and storage practices, opinions on firearm injury prevention strategies, and perceptions of safety among adults in California households with and without children and/or adolescents.

**Design, Setting, and Participants:**

This survey study used data from the 2018 California Safety and Well-being Survey, a California-representative, probability-based internet survey. Respondents were part of the Ipsos KnowledgePanel, an online research panel that uses address-based sampling and provides survey weights to produce estimates representative of the adult population of California.

**Main Outcomes and Measures:**

Topics included firearm storage practices, opinions on interventions to reduce pediatric firearm injury, and perceptions of household safety related to firearm ownership. Respondents were stratified by firearm ownership and household presence or absence of children and/or adolescents. Weighted percentages and 95% CIs are presented.

**Results:**

Of 5232 invited panel members, 2558 (48.9%) completed the survey. Among respondents, 52.5% (95% CI, 49.3%-55.7%) were women, 42.9% (95% CI, 39.9%-45.9%) were White, 30.0% (95% CI, 26.8%-32.9%) lived in homes with children, and the mean (SD) age was 48.0 (17.1) years. Among those in homes with children, more than two-thirds of individuals who owned firearms (70.6% [95% CI, 50.1%-85.2%]) and more than half of individuals who did not own firearms but lived in homes with guns (54.9% [95% CI, 37.9%-70.8%]) reported that they believed a firearm in the home made it safer. Half of those who owned firearms (52.3% [95% CI, 34.9%-69.2%]) and more than three-quarters of individuals who did not own firearms but lived in homes with guns (78.4% [95% CI, 57.5%-90.7%]) reported it was always appropriate for parents to inquire about unlocked guns in homes where their children play. Among those who had previously owned at least 1 gun but no longer did, 13.3% (95% CI, 7.1%-23.8%) reported getting rid of guns at least in part due to concern for the safety of a child in the home. Nearly two-thirds of those who owned firearms living with children and/or adolescents (64.5% [95% CI, 46.5%-79.2%]) did not store all firearms in the most secure manner (ie, unloaded and locked up), compared with 36.4% (95% CI, 29.4%-44.1%) of individuals who owned firearms but did not live with children.

**Conclusions and Relevance:**

In this study, although a substantial percentage of individuals who owned guns and lived with children did not store all firearms as recommended, parents and caregivers who owned firearms reported being amenable to interventions that reduce young people’s risk of firearm-related harm. Future work should investigate acceptable risk reduction and safe storage interventions.

## Key Points

**Question:**

Are there differences in firearm storage practices, opinions on firearm injury prevention strategies, and perceptions of safety among California adults in homes with and without children and/or adolescents?

**Findings:**

In this survey of 2558 adults in California, respondents who owned firearms and lived with children and/or adolescents reported higher rates of storing guns loaded and/or unlocked than those living in homes without children. The survey found high support among California adults, including among those who owned guns, for conversations with health professionals and other parents and caregivers designed to keep children safer from firearm injury.

**Meaning:**

These findings suggest that most California households with children and/or adolescents and guns do not store all firearms unloaded and locked up, but that parents and caregivers may be amenable to interventions focused on changing storage practices to keep children and adolescents safer.

## Teaser

This survey study compares firearm ownership and storage practices, opinions on firearm injury prevention strategies, and perceptions of safety among adults in California households with and without children and/or adolescents.

## Introduction

Firearm injury is consistently among the leading causes of death for children and adolescents in the United States. In 2019, although homicide accounted for most of the 3390 firearm deaths (59.9%) among individuals aged 0 to 19 years, more than one-third of deaths (34.4%) were from firearm suicide, and 3.5% were from unintentional firearm injuries.^1^ Moreover, suicide rates among adolescents have increased more than those for any other age group in the last decade.^1,2,3^

Firearms in the home increase the risk of firearm-related harm.^4,5,6,7,8,9^ Storing all firearms unloaded and locked up using a locking device can reduce the risk of unintentional firearm injury and firearm suicide for children and adolescents.^10^ Nationally, fewer than one-third of individuals who own guns and live in households with children report storing all guns locked up and unloaded (the manner recommended by the American Academy of Pediatrics).^11^ This may be due in part to misperceptions about risk. Research suggests that most adults in households with children, especially those with firearms in the home, do not associate household firearms with increased risk of suicide.^12^ Discordance between parent and child reports of child firearm access is also common. A 2019 national survey found that 45% of parents who did not own guns and one-third of parents who owned guns and said their adolescent child could not access a household firearm were contradicted by their child’s own report of access.^13^

National surveys have examined firearm ownership and opinions about firearm policies in households with children and adolescents,^11^ but data at the state level have not been available. This is a critical gap in knowledge, as firearm ownership and the cultural acceptability of firearms, firearm-related harm, and firearm regulations vary markedly by state.^14,15^ New firearm injury prevention policies and practices are most often developed and enacted by states and localities. This study can inform the development of targeted, acceptable, and appropriate education and interventions for those living in homes with children and/or adolescents to reduce pediatric firearm injury.

In this state-representative survey study, we compared perceptions of risk associated with firearms in the home and opinions on firearm injury prevention strategies among a representative sample of adults in California households (one-quarter of which have a firearm),^14^ stratifying by the presence of children or adolescents and by the presence of firearms. We provide estimates of the percentage of adults who have disposed of firearms out of concern for the safety of children in the home and of the appropriateness of parents/caregivers asking about firearms in homes where their children go to play. We then compared characteristics of firearm ownership, reasons for firearm ownership, and firearm storage practices among those who own firearms and live in homes with and without children and/or adolescents.

## Methods

We analyzed data from the 2018 California Safety and Well-being Survey (CSaWS), a probability-based internet survey designed by the authors and administered online by Ipsos (formerly the GfK Group).^16^ Survey respondents were drawn from the Ipsos KnowledgePanel, a national online panel of 55 000 randomly recruited adult members that has been widely used in health-related research.^11,15,17,18,19^ KnowledgePanel included approximately 6700 California adults at the time of survey administration. Recruitment into the panel is continuous through probability-based sampling of the US Postal Service’s Delivery Sequence File. The sampling frame is augmented by disproportionate stratified sampling of households with younger members (ie, 18-29 years) and Latinx residents.

A study-specific sample was selected using probability proportional to size procedures based on geodemographic benchmarks from the US Census Current Population Survey. All panel members aged 18 years and older who lived in California and were not on active duty in the US armed forces were eligible. The survey was available in English and Spanish. Eligible panel members were emailed invitations to complete the survey, and nonresponders were automatically emailed reminders. Ipsos provides web-enabled devices and internet access to panel households as needed. No study-specific incentives were provided. Further details on the study sample methodology have been published previously.^14^

Participants read informed consent language, and their initiation of the survey constituted consent. This study followed the reporting guideline of the American Association for Public Opinion Research (AAPOR). The survey was approved by the University of California Davis institutional review board.

### Measures

We asked about household firearms and personal firearm ownership to ascertain respondents’ ownership status. Respondents were categorized as follows: those who owned a firearm, those who did not own firearms but lived in homes with firearms, and those who did not own or live with firearms (ie, “no firearms in household”). All respondents were asked for their opinions on specific interventions to reduce risk of firearm injury in children and/or adolescents with the following 3 questions: (1) “In general, how often is it appropriate for doctors and other health professionals to talk to their patients about gun safety?”; (2) “In general, how often is it appropriate for doctors or other health professionals to talk to patients about gun safety when the patient has guns in the home and lives with children or teens?”; and (3) “How often is it appropriate for parents to ask if there are unlocked guns in a home where their children go to play?” All respondents were also asked whether they feel a gun in their home does or would make their home a safer or more dangerous place to be.

Those who owned firearms were asked what types of firearms they owned, how many of each type, and primary reasons for ownership. They were also asked whether they keep their firearms loaded or unloaded and locked up or not locked up. Respondents who lived in homes without firearms or who reported that they had “gotten rid of any gun(s)” were asked why they did not currently own a gun or have a gun in the home. Full question text is available in the eAppendix in the Supplement.

Respondent sociodemographic variables, including age, race/ethnicity, sex, marital status, household income, education, ages of children and/or adolescents younger than 18 years in the home (hereafter “children”), region and rurality of residence, and veteran status, were provided by Ipsos. Race/ethnicity was self-reported and included non-Latinx White, non-Latinx Black, non-vmultiracial, other non-Latinx race (ie, American Indian or Alaska Native, Asian, Native Hawaiian or Pacific Islander, or “some other race”), and Latinx. Data were collected on entry into the panel but are of interest for this study to examine differences in the prevalence of gun ownership by race/ethnicity in homes with and without children

### Statistical Analysis

We calculated weighted percentages and 95% CIs for all tabulations using Stata version 15.1 (StataCorp) and the svy suite of commands. The survey weighting variable was provided by Ipsos and combines presample and study-specific poststratification weights. Presample weights adjust for the probability of selection into the panel and discrepancies between the distribution of key demographic characteristics in the US population (as per the latest supplement of the US Census Current Population Survey) and the panel members. Poststratification weights account for survey nonresponse and over- or underrepresentations of key demographic characteristics between California’s adult population and survey respondents by using raking ratio adjustments based on cross-classifications of key demographic variables. Resulting estimates are statistically representative of the adult population of the state.

## Results

Ipsos invited 5232 panel members to participate in CSaWS, and 2558 (48.9%) completed the survey. Firearm owning status information was provided by 2468 respondents (96.5%). Among respondents in this analytic sample, 52.5% (95% CI, 49.3%-55.7%) were women, 42.9% (95% CI, 39.9%-45.9%) were White, and the mean (SD) age was 48.0 (17.1) years. Overall, 30.0% (95% CI, 26.8%-32.9%) of respondents lived in homes with children. Additional sociodemographic characteristics of respondents appear in Table 1 and Table 2. Compared with panel members who did not respond to the survey, survey respondents were more often older, male, non-Latinx, did not have children at home, had higher income, and had more years of education.^14^

**Table 1.** Sociodemographic Characteristics of Respondents With Children and/or Adolescents in the Household by Firearm Ownership Status^a^

Characteristic	Full sample, % (95% CI) (N = 2468)	Respondents living with children and/or adolescents				
		Total, No.	Total, % (95% CI)	Owned firearms, % (95% CI)	Lived in household with firearm(s), % (95% CI)	No firearms in household, % (95% CI)
Total	NA	522	NA	9.2 (6.4-12.9)	12.1 (8.6-16.8)	78.7 (73.4-83.2)
Age, y						
18-29	17.7 (14.9-20.9)	40	17.4 (12.6-23.7)	3.6 (0.5-21.3)	43.2 (26.3-61.9)	15.1 (10.0-22.1)
30-44	27.4 (24.5-30.4)	234	47.2 (41.0-53.5)	50.1 (32.8-67.3)	34.1 (20.8-50.5)	48.9 (41.8-56.0)
45-59	27.1 (24.5-30.0)	191	30.2 (25.0-36.0)	44.1 (27.7-61.9)	20.4 (10.1-36.7)	30.1 (24.3-36.8)
≥60	27.8 (25.4-30.2)	57	5.1 (3.3-7.8)	2.3 (0.8-6.6)	2.3 (0.7-7.1)	5.9 (3.7-9.3)
Sex						
Male	47.5 (44.3-50.7)	173	39.7 (33.6-46.1)	66.1 (48.9-79.9)	29.3 (14.5-50.3)	38.2 (31.3-45.5)
Female	52.5 (49.3-55.7)	349	60.3 (53.9-66.4)	33.9 (20.1-51.1)	70.7 (49.7-85.5)	61.8 (54.5-68.7)
Race/ethnicity						
Non-Latinx						
White	42.9 (39.9-45.9)	186	29.3 (24.6-34.6)	51.7 (34.1-69.0)	39.2 (24.1-56.7)	25.2 (20.3-30.8)
Black	5.5 (4.3-7.2)	31	5.5 (3.5-8.5)	9.8 (1.8-38.9)	8.8 (3.7-19.5)	4.5 (2.8-7.1)
Other^b^	16.0 (13.6-18.7)	42	15.8 (11.5-21.4)	2.4 (0.3-15.3)	6.0 (1.5-21.3)	18.9 (13.6-25.7)
Multiracial	2.4 (1.7-3.5)	12	3.1 (1.5-6.3)	6.1 (1.5-21.6)	6.4 (0.9-33.6)	2.2 (1.0-5.0)
Latinx	33.2 (30.0-36.5)	251	46.2 (40.0-52.6)	30.0 (15.9-49.1)	39.5 (23.7-58.0)	49.2 (42.1-56.3)
Education						
<High school	9.1 (7.1-11.7)	49	12.6 (8.6-18.0)	0.0	1.4 (0.2-9.2)	15.8 (10.9-22.4)
High school	24.6 (21.6-27.7)	66	24.6 (19.2-31.1)	22.8 (9.5-45.4)	30.9 (15.4-52.2)	23.9 (17.9-31.1)
Some college	32.7 (29.8-35.6)	170	30.1 (24.8-36.1)	43.6 (27.3-61.5)	40.1 (24.6-57.8)	27.0 (21.2-33.8)
Bachelor’s degree	18.6 (16.6-20.9)	145	18.9 (15.0-23.5)	21.5 (11.3-37.1)	17.5 (9.2-30.6)	18.8 (14.3-24.3)
Advanced degree	15 (13.2-17.1)	92	13.7 (10.5-17.8)	12.0 (5.5-24.1)	10.2 (4.9-20.0)	14.5 (10.7-19.4)
Political party						
Republican	19 (16.8-21.3)	83	12.8 (9.6-16.9)	31.7 (18.5-48.8)	30.4 (16.3-49.4)	7.9 (5.4-11.3)
Democrat	40.6 (37.5-43.8)	206	41.0 (34.9-47.3)	30.5 (15.5-51.1)	42.5 (26.4-60.3)	42.0 (35.0-49.2)
No preference	33.9 (30.9-37.1)	203	41.5 (35.5-47.8)	33.6 (19.5-51.2)	27.2 (14.9-44.3)	44.6 (37.6-51.8)
Other	5.9 (4.6-7.5)	26	4.3 (2.6-7.1)	4.2 (0.6-24.5)	0.0	4.9 (2.9-8.3)
Guns in the home growing up						
No	60.6 (57.5-63.6)	312	65.5 (59.4-71.1)	40.9 (25.0-59.0)	46.1 (29.4-63.6)	71.3 (64.5-77.3)
Yes	34.1 (31.3-37.1)	182	26.6 (21.8-32.0)	57.7 (39.8-73.8)	44.7 (28.4-62.2)	20.2 (15.6-25.6)
Do not know	4.7 (3.4-6.3)	25	6.2 (3.4-10.9)	1.4 (0.2-9.5)	9.2 (2.6-27.7)	6.3 (3.1-12.1)
Urbanicity of residence						
Urban	91.3 (89.5-92.9)	468	92.4 (88.6-95.0)	80.0 (63.5-90.2)	93.6 (82.4-97.8)	93.7 (89.1-96.4)
Suburban or rural	8.7 (7.1-10.5)	47	7.6 (5.0-11.4)	20.0 (9.8-36.5)	6.4 (2.2-17.6)	6.3 (3.6-10.9)
Veteran	8.7 (7.4-10.3)	30	3.9 (2.4-6.5)	13.6 (5.9-28.4)	0.3 (0.0-2.1)	3.4 (1.8-6.4)
Ages of child(ren) in household, y						
0-5	11.2 (9.2-13.6)	178	37.8 (31.8-44.1)	33.0 (18.5-51.7)	32.9 (19.4-49.9)	39.1 (32.2-46.4)
6-12	15.4 (13.1-18.1)	269	51.8 (45.6-58.0)	67.2 (50.1-80.7)	61.3 (43.2-76.7)	48.6 (41.5-55.7)
13-17	15.6 (13.4-18.2)	268	52.5 (46.3-58.7)	45.9 (29.2-63.5)	46.5 (29.9-63.9)	54.2 (47.0-61.2)

Abbreviation: NA, not applicable.

^a^ Some columns do not total to 100% because those who refused the question are excluded. All percentages are weighted, and counts are unweighted.

^b^ Respondents in the other category were those who selected American Indian or Alaska Native, Asian, Native Hawaiian or Pacific Islander, or “some other race.”

**Table 2.** Sociodemographic Characteristics of 1946 Respondents Without Children and/or Adolescents in the Household by Firearm Ownership Status^a^

Characteristic	Full sample (N = 2468)	Respondents not living with children and/or adolescents				
		Total, No.	Total, % (95% CI)	Owned firearms, % (95% CI)	Lived in household with firearm(s), % (95% CI)	No firearms in household, % (95% CI)
Total	NA	1946	NA	17.4 (15.0-20.1)	10.5 (8.4-13.0)	72.1 (68.8-75.2)
Age						
18-29	17.7 (14.9-20.9)	109	17.8 (14.5-21.7)	7.1 (3.1-15.4)	25.9 (15.3-40.3)	19.3 (15.3-24.0)
30-44	27.4 (24.5-30.4)	227	19.0 (16.1-22.3)	10.2 (6.2-16.3)	16.1 (9.0-27.0)	21.5 (17.9-25.7)
45-59	27.1 (24.5-30.0)	462	25.8 (22.8-29.1)	30.7 (23.4-39.1)	24.3 (16.3-34.7)	24.8 (21.4-28.7)
≥60	27.8 (25.4-30.2)	1148	37.4 (34.2-40.6)	52.0 (44.0-59.8)	33.7 (25.1-43.6)	34.3 (30.8-38.1)
Sex						
Male	47.5 (44.3-50.7)	870	50.8 (47.1-54.5)	74.5 (68.0-80.0)	25.9 (15.5-40.0)	48.7 (44.3-53.1)
Female	52.5 (49.3-55.7)	1076	49.2 (45.5-52.9)	25.5 (20.0-32.0)	74.1 (60.0-84.5)	51.3 (46.9-55.7)
Race/ethnicity						
Non-Latinx						
White	42.9 (39.9-45.9)	1213	48.6 (45.0-52.2)	66.8 (58.3-74.4)	62.8 (50.5-73.6)	42.1 (38.1-46.3)
Black	5.5 (4.3-7.2)	86	5.6 (4.0-7.6)	3.2 (1.8-5.7)	5.2 (1.5-16.4)	6.2 (4.3-8.8)
Other^b^	16.0 (13.6-18.7)	140	16.1 (13.4-19.3)	10.5 (5.8-18.5)	9.7 (4.2-20.8)	18.4 (15.0-22.3)
Multiracial	2.4 (1.7-3.5)	42	2.1 (1.4-3.1)	1.2 (0.4-3.1)	4.9 (1.9-12.1)	1.9 (1.2-3.1)
Latinx	33.2 (30.0-36.5)	465	27.6 (24.1-31.5)	18.3 (12.2-26.4)	17.3 (9.7-29.0)	31.4 (27.0-36.1)
Education						
<High school	9.1 (7.1-11.7)	60	7.7 (5.5-10.7)	3.3 (1.0-10.3)	3.1 (1.1-8.4)	9.4 (6.5-13.4)
High school	24.6 (21.6-27.7)	229	24.5 (21.1-28.2)	23.0 (16.4-31.2)	34.8 (23.4-48.2)	23.4 (19.5-27.8)
Some college	32.7 (29.8-35.6)	682	33.7 (30.5-37.2)	41.9 (34.5-49.7)	38.1 (28.0-49.4)	31.1 (27.3-35.2)
Bachelor's degree	18.6 (16.6-20.9)	559	18.5 (16.3-21.1)	18.1 (13.2-24.3)	13.8 (9.1-20.5)	19.3 (16.6-22.4)
Advanced degree	15.0 (13.2-17.1)	416	15.5 (13.4-18.0)	13.7 (9.9-18.7)	10.2 (6.0-16.6)	16.8 (14.0-19.9)
Political party						
Republican	19.0 (16.8-21.3)	477	21.6 (18.9-24.5)	39.7 (32.3-47.6)	20.7 (13.6-30.4)	17.3 (14.5-20.5)
Democrat	40.6 (37.5-43.8)	825	40.5 (36.9-44.1)	28.0 (21.4-35.6)	32.8 (23.8-43.2)	44.6 (40.3-49.0)
No preference	33.9 (30.9-37.1)	504	30.7 (27.3-34.4)	26.1 (19.7-33.7)	34.3 (23.5-47.1)	31.3 (27.2-35.7)
Other	5.9 (4.6-7.5)	125	6.6 (5.0-8.6)	5.9 (3.7-9.3)	12.0 (5.7-23.6)	6.0 (4.3-8.3)
Guns in the home growing up						
No	60.6 (57.5-63.6)	1060	58.5 (54.9-62.0)	22.4 (16.8-29.2)	44.3 (33.4-55.8)	69.3 (65.3-73.0)
Yes	34.1 (31.3-37.1)	805	37.3 (33.9-40.9)	73.8 (66.9-79.8)	47.5 (36.2-59.0)	27.1 (23.5-30.9)
Do not know	4.7 (3.4-6.3)	77	4.0 (2.9-5.6)	3.6 (1.9-6.6)	8.0 (3.2-18.7)	3.6 (2.4-5.2)
Urbanicity of residence						
Urban	91.3 (89.5-92.9)	1728	90.9 (88.7-92.7)	84.3 (78.9-88.6)	84.7 (74.0-91.5)	93.4 (91.0-95.2)
Suburban or rural	8.7 (7.1-10.5)	203	9.1 (7.3-11.3)	15.7 (11.4-21.1)	15.3 (8.5-26.0)	6.6 (4.8-9.0)
Veteran	8.7 (7.4-10.3)	282	10.8 (9.0-12.8)	32.7 (26.0-40.1)	0.7 (0.1-2.9)	6.9 (5.4-8.9)

Abbreviation: NA, not applicable.

^a^ Some columns do not total to 100% because those who refused the question are excluded. All percentages are weighted and counts are unweighted.

^b^ Respondents in the other category were those who selected American Indian or Alaska Native, Asian, Native Hawaiian or Pacific Islander, or “some other race.”

### Households With Children and Firearms

One-quarter of respondents (25.9%; 95% CI, 23.3%-29.0%) reported living in a home with 1 or more firearms, including 21.3% (95% CI, 16.8%-26.6%) of those with children in the household (Table 1) and 27.9% (95% CI, 24.8%-31.2%) without children (Table 2). Those who owned firearms and lived in households with children were more often older and male compared with the total population of respondents. Although a plurality of all respondents in households with children were Latinx individuals (46.2%; 95% CI, 40.0%-52.6%), most individuals who owned firearms and lived in homes with children were White, non-Latinx (51.7%; 95% CI: 34.1%-69.0%). Those who owned firearms and lived in households with children disproportionately lived in suburban or rural areas compared with the proportion of all households with children.

### Perceptions of Safety

Respondents in homes with firearms more frequently reported that firearms make homes safer compared with those without firearms in the home, regardless of the presence of children (Table 3). The percentage of respondents who agreed that firearms in the home made their home safer ranged from 8.6% (95% CI, 5.7-12.6) among those in homes without children or guns to 70.6% (95% CI, 50.1%-85.2%) among those in households with guns and children (Table 3). More than half of individuals who did not own firearms but lived in homes with guns (54.9% [95% CI, 37.9%-70.8%]) reported that having a firearm in the home made it safer.

**Table 3.** Perceived Effect of Having a Gun in the Home on Home Safety, by Firearm Ownership and Presence of Children in the Household

Response	Respondents, % (95% CI)					
	Owned firearms		Lived in household with firearm(s)		No firearms in household	
	No children in household (n = 377)	Children in household (n = 48)	No children in household (n = 179)	Children in household (n = 63)	No children in household (n = 1384)	Children in household (n = 410)
Safer	58.7 (50.8-66.2)	70.6 (50.1-85.2)	47.0 (35.7-58.7)	54.9 (37.9-70.8)	16.7 (13.4-20.3)	8.6 (5.7-12.6)
More dangerous	5.4 (2.9-9.9)	0	5.6 (2.5-12.2)	4.5 (1.7-11.5)	34.7 (30.7-38.9)	44.2 (37.3-51.4)
It depends	21.4 (15.1-29.5)	22.2 (9.4-44.0)	17.5 (11.6-25.5)	11.1 (5.1-2.3)	17.0 (13.9-20.6)	15.6 (11.0-21.5)
Do not know	14.4 (10.3-19.8)	7.2 (2.0-22.5)	30.0 (20.1-41.9)	29.6 (17.2-4.6)	31.2 (27.7-36.1)	31.7 (25.2-38.9)

### Opinions on Firearm Injury Prevention Strategies

Among respondents living in households with children, support for 2 firearm injury prevention strategies was generally high, regardless of firearm ownership status (Table 4). Individuals who did not own guns but lived in households with guns and children more often reported that parents asking about unsecured guns in other people’s homes where their children play was always appropriate (78.4%; 95% CI, 57.5%-90.7%) compared with those who owned guns and lived in households with children (52.3%; 95% CI, 34.9%-69.2%) and those in no-firearm households with children (49.8%; 95% CI, 42.7%-56.9%). Similar percentages of respondents without children in the home found such conversations at least sometimes appropriate (results not shown).

**Table 4.** Opinions on Firearm Injury Prevention Strategies in Households With Children by Firearm Ownership Status^a^

Response	Total respondents, No.	% (95% CI)			
		Total respondents	Owned firearms (n = 49)	Lived in households with firearms (n = 63)	No firearms in household (n = 410)
**How often is it appropriate for parents to ask if there are unlocked guns in a home where their children go to play?**					
Never	47	6.2 (3.9-9.7)	9.4 (3.5-22.7)	0.8 (0.1-4.6)	6.7 (4.0-11.0)
Sometimes or usually	122	25.0 (20.0-30.8)	32.0 (17.7-50.6)	5.0 (2.3-10.9)	27.3 (21.4-34.1)
Always	291	53.5 (47.2-59.7)	52.3 (34.9-69.2)	78.4 (57.5-90.7)	49.8 (42.7-56.9)
**In general, how often is it appropriate for doctors and other health professionals to talk to their patients about gun safety?**					
Never	62	10.2 (7.2-14.2)	43.8 (27.6-61.4)	6.8 (2.7-15.8)	6.8 (4.0-11.4)
Sometimes or usually	155	27.5 (22.5-33.2)	15.7 (7.8-29.1)	48.0 (31.0-65.5)	25.7 (20.4-31.8)
Always	256	50.4 (44.2-56.6)	36.9 (20.9-56.5)	36.2 (22.3-52.8)	54.2 (47.1-61.1)
**In general-how often is it appropriate for doctors or other health professionals to talk to patients about gun safety when the patient has guns in the home and lives with children or teens?**					
Never	40	6.0 (3.7-9.6)	17.8 (7.8-35.9)	1.5 (0.4-5.1)	5.3 (2.9-9.6)
Sometimes or usually	106	20.3 (15.8-25.9)	25.2 (13.4-42.1)	39.6 (23.1-58.8)	16.8 (12.2-22.6)
Always	337	66.3 (60.1-72.0)	38.4 (23.2-56.2)	54.5 (36.4-71.5)	71.3 (64.5-77.3)

^a^ All percentages are weighted and all counts are unweighted. Percentages do not add to 100 because proportions who responded “do not know” are not included.

Most respondents in homes with children indicated support for health professionals talking with their patients about gun safety in general (78.1%; 95% CI, 72.5%-82.8%) and when the patient “lives with children or teens” (86.7%; 95% CI, 81.8%-90.4%). Support varied by firearm ownership status (Table 4); 17.8% (95% CI: 7.8%-35.9%) of those who owned firearms said health professionals talking with patients about gun safety was “never” appropriate when the patient lives with children or adolescents.

### Reasons for Ownership, Firearms Owned, and Storage Practices

Among those who owned firearms, self-protection was cited most commonly as a reason for ownership in homes with and without children (77.3% [95% CI, 60.7%-88.3%] and 63.6% [95% CI, 55.8%-70.7%], respectively) (Table 5). Those who owned guns and lived in homes with children tended to own fewer guns than those in homes without children.

**Table 5.** Firearms Owned, Reasons for Ownership, and Firearm Storage Methods Among Respondents Who Owned Firearms by Presence of Children in the Household^a^

Response	All respondents		Children in home, % (95% CI)	
	No. (n = 529)	% (95% CI)	No (n = 380)	Yes (n = 49)
Owned ≥1 firearm for				
Self-protection	281	66.1 (59.1-72.5)	63.6 (55.8-70.7)	77.3 (60.7-88.3)
Hunting	104	26.0 (19.9-33.3)	28.2 (21.2-36.4)	16.1 (7.1-32.4)
Sporting use	188	46.7 (39.6-53.9)	47.4 (39.6-55.3)	43.6 (27.5-61.3)
Collecting	112	24.7 (19.2-31.2)	25.2 (19.2-32.4)	22.3 (11.3-39.2)
Inheritance	27	4.8 (2.9-7.7)	3.6 (2.2-5.9)	10.1 (3.6-24.8)
Firearms owned, No.				
1-2	165	38.0 (31.4-45.1)	50.8 (42.9-58.7)	70.0 (52.8-83.0)
≥3	143	34.4 (27.7-41.7)	49.2 (41.3-57.1)	30.0 (17.1-47.2)
Type of firearms owned				
Handguns	344	81.4 (74.9-86.5)	81.3 (74.2-86.8)	81.7 (62.3-92.3)
Long guns	291	69.1 (62.4-75.0)	72.3 (65.5-78.2)	54.5 (36.6-71.4)
Storage methods^b^				
Any gun unlocked and loaded	78	19.8 (14.0-27.2)	23.1 (16.3-31.7)	4.0 (0.9-15.8)
No guns unlocked and loaded but ≥1 loaded and locked or unloaded and unlocked	176	41.4 (34.3-48.9)	36.4 (29.4-44.1)	64.5 (46.5-79.2)
All guns stored unloaded and locked	133	38.9 (31.7-46.6)	40.5 (32.5-48.9)	31.5 (17.8-49.3)

^a^ All percentages are weighted and all counts are unweighted.

^b^ A total of 32 respondents stored some firearms outside the home and, due to a questionnaire programming error, could not be included in this calculation.

Individuals who owned guns and lived in households with children less often reported storing any gun unlocked and loaded (the least secure manner) compared with owners in homes without children (4.0% [95% CI,0.9-15.8] vs 23.1% [95% CI: 16.3-31.7]) (Table 5). Nearly two-thirds of those who owned firearms living with children and/or adolescents (64.5% [95% CI, 46.5%-79.2%]) did not store all firearms in the most secure manner (ie, unloaded and locked up), compared with 36.4% (95% CI, 29.4%-44.1%) of individuals who owned firearms but did not live with children. However, individuals who owned guns and lived in households with children reported storing all firearms in the recommended manner (ie, unloaded and locked up, with a trigger lock, cable lock, in a lock box or safe, or in some other way) at the same rate as those living in households without children.

### Reasons for Not Owning Firearms

Among respondents who had never owned a gun (n = 1843), 1 in 4 (23.6% [95% CI, 20.6%-26.8%]) said this was at least in part due to concern for the safety of children in the home. Among respondents who had owned a gun in the past but not at the time of the survey (n = 270), 13.3% (95% CI, 7.1%-23.8%) said they had stopped owning guns at least in part due to concern for the safety of a child in the home. Most of those who disposed of guns out of concern for a child in the home (85.2% [95% CI, 56.7%-96.2%]) had not owned a gun in the last 5 years.

## Discussion

Our findings indicate that approximately 1 in 5 (21%) California households with children, or 931 000 households, contains at least 1 firearm. Research shows that a young person’s access to firearms increases risk of unintentional shootings and firearm suicide.^4,5,10,20,21,22,23,24,25^ The likelihood of unauthorized firearm access by children is greater when firearms are not stored securely. For this reason, major medical and pediatric professional societies recommend that all guns in homes with children are stored locked up and unloaded to reduce the possibility of children gaining unauthorized access to a firearm and using it.^26,27^

Nationally, 21% of gun-owning households with children store at least 1 firearm in the least secure manner, ie, approximately 4.6 million children live in a home with at least 1 loaded and unlocked gun.^11^ This percentage was substantially lower in California; just 4% of gun-owning households with children—an estimated 37 000 households—reported storing at least 1 firearm loaded and not locked up. Of note, California is among the 15 states and the District of Columbia that impose criminal penalties for negligent firearm storage (via child access prevention [CAP] laws), holding those who own guns liable when they know or reasonably should know that a child’s access to a firearm is likely, whether loaded or unloaded, and regardless of whether the minor actually gains access to the firearm.^28^ The evidence on the effectiveness of CAP laws is limited, however.^29,30^ Future research should examine the association between CAP laws and storage practices among those who own firearms and live with children.

Nonetheless, only one-third (32%) of gun-owning California households with children reported storing all firearms in the recommended manner; most (65%; an estimated 600 000 households) reported using some intermediate type of storage—ie, no guns stored loaded and unlocked but at least 1 gun stored either loaded and locked up or unloaded and not locked up. Parents and caregivers may not be aware that children in their home have access to firearms, and educational campaigns designed to teach children to stay away from firearms have been shown not to be effective.^31,32,33^

Campaigns promoting conversations about the importance of safe firearm storage are ongoing.^34^ We found widespread support for parents and/or caregivers asking about firearms in homes where their children play: more than three-quarters of respondents with and without children in the home, regardless of firearm ownership status, reported that these conversations were at least sometimes appropriate. This novel finding suggests that among California adults, asking questions about whether firearms are kept inaccessible to children who spend time in others’ homes may be well received.

Similarly, clinician-initiated conversations about safe firearm storage with parents and caregivers of children may help to promote keeping firearms unloaded and locked up.^35,36^ Our findings indicate that most California residents who live with children think it is at least sometimes appropriate for health professionals to talk about gun safety. This includes more than half of respondents who owned firearms (compared with 52%-65% of those who own guns nationwide, depending on the age of the child in the home) and more than 80% of those who do not own firearms but live in homes with them (compared with 65%-74% of such respondents nationwide, depending on the age of the child).^17^ Additional research examining tailored and culturally-relevant firearm safe storage messaging and storage methods is warranted.^37^

This study adds to the literature, as it found increased support for such conversations when a patient lives with children or adolescents, bolstering the recommendation that clinicians establish context when discussing access to firearms, risk of injury, and prevention strategies. Experts recommend efforts that go beyond guidance and use more active methods, such as conversations on safe firearm storage tailored to the patient or household, to prevent firearm injury in children.^11,38^

We also compared perceptions of safety among households with and without children across firearm ownership status groups, building on national survey research, which estimates that 58% of firearm owners think the presence of guns makes their home safer and that these owners more often store at least 1 gun loaded and unlocked.^39^ In our study, the presence of children in a home did not seem to affect perceived safety: more respondents in households with guns and children thought a firearm made their home safer compared with households with guns but without children. It is possible that this finding reflects the question posed, which did not define a type of hypothetical risk (whether in the home, such as suicide, or from outside of the home, such as an intruder). Nevertheless, this further suggests that adults in homes with firearms may not perceive the increased risk of firearm-related harm.^12^ Messaging about the increased risk of firearm injury for those in households with firearms and the importance of safe storage to reduce risk may help to reduce firearm-related harm.

Our results suggest that some California adults are aware of the increased risk of a firearm in homes where children live, as notable proportions of respondents cited concern for the safety of children in the home as a reason to not own guns or a reason for those who owned firearms to dispose of them. Little is known on this topic, and future research should investigate what drives firearm those who own to stop owning firearms.

California firearm injury prevention policies and practices are sometime used as models for other states, including most recently with extreme risk protection order laws. State-specific findings such as these may inform those developing policies and practices on gaps and amenability to pediatric injury prevention strategies.

### Limitations

This study has limitations. As with all survey studies, social desirability and nonresponse biases are possible. Panel members who chose to participate in our study may be different than those who chose not to participate; nonresponders tended to be younger, Latinx, female; to have lower income and fewer years of education; and to live in homes with children compared with our survey’s respondents. Our sample size was relatively small, as was the proportion of respondents who owned firearms, limiting our ability to conduct additional subgroup analyses. Because our survey was among respondents in a single state—a state with relatively low rates of firearm ownership and relatively restrictive firearm policies—these results may not be generalizable to other states or the nation as a whole.

## Conclusions

Findings from this analysis of data from a state-representative survey of adults in California underscore the continued need for messaging on the risk of firearm access and the importance of safe storage. This study may inform ongoing efforts to reduce firearm-related harm in homes with children and adolescents. Adults in California, including those who owned guns, were supportive of interventions to prevent childhood firearm injury, especially parents/caregivers asking about firearms in homes where their children play.

## Notes

Supplement. **eAppendix.** Question Text and Response Options
