# Error in Title

**DOI:** 10.1001/jamanetworkopen.2021.27740

**Published:** 2021-08-26

In the Original Investigation titled “Firearm Practices, Perceptions of Safety, and Opinions on Injury Prevention Strategies Among California Adults With vs Without Children,”^1^ published August 3, 2021, the phrase “with vs without children” was not included in the title. This article has been corrected.^1^
