# Supplementary material for: Firearm Practices, Perceptions of Safety, and Opinions on Injury Prevention Strategies Among California Adults
Source: JAMA Netw Open. 2021 Aug 3;4(8):e2119146. doi: 10.1001/jamanetworkopen.2021.19146 (PMC8335574; doi:10.1001/jamanetworkopen.2021.19146)
Supplement: Supplement. — eAppendix. Question Text and Response Options [file jamanetwopen-e2119146-s001.pdf]

Supplemental Online Content

Pallin R, Wintemute GJ, Kravitz-Wirtz N. Firearm practices, perceptions of safety, and opinions on injury prevention strategies among California adults. *JAMA Netw Open.* 2021;4(8):e2119146. doi:10.1001/jamanetworkopen.2021.19146

eAppendix. Question Text and Response Options

This supplemental material has been provided by the authors to give readers additional information about their work.

© 2021 Pallin R et al. *JAMA Network Open.*

Question text and response options as presented in 2018 California Safety and Wellbeing Survey (CSaWS) included in analysis for "Firearm practices, perceptions of safety, and opinions on injury prevention strategies in California homes with children"

Note: Respondent demographic information is collected by Ipsos upon recruitment into the panel and provided with the survey data. This includes information on respondent age, gender, race/ethnicity, urbanicity of residence, education, income, marital status, presence of children in the home, and military veteran status.

Do you or does anyone else you live with currently own any type of gun?
- Yes
- No
- Don't know

Do you personally own a gun?
- Yes
- No

When you were growing up, were there ever any guns kept in your home?
- Yes
- No
- Don't know

Which of the following types of guns do you own?
- Handguns
- Long guns such as rifles or shotguns

How many handguns do you own?

How many of these handguns are…?
- Semi-automatic pistols
- Revolvers
- Other

Please choose the reason(s) you own handgun(s).
- For protection against people
- For protection against animals
- For hunting
- For sporting use (other than hunting)
- For a collection
- For some other reason

Please choose the one most important reason you own handgun(s).
- For protection against people
- For protection against animals
- For hunting
- For sporting use (other than hunting)
- For a collection
- For the other reason you mentioned

How many long guns do you own?

How many of these long guns are…?
- Semi-automatic rifles
- Rifles of other types
- Semi-automatic shotguns

© 2021 Pallin R et al. *JAMA Network Open.*

Shotguns of other types

Please choose the reason(s) you own long gun(s).
For protection against people
For protection against animals
For hunting
For sporting use (other than hunting)
For a collection
For some other reason

How do you usually store your [handgun(s)/long gun(s)]?
Unloaded and locked up (with a trigger lock, cable lock, in a lock box or safe, or in some other way)
Unloaded and not locked up
Loaded and locked up
Loaded and not locked up

Please choose the one most important reason you own long gun(s).
For protection against people
For protection against animals
For hunting
For sporting use (other than hunting)
For a collection
For the other reason you mentioned

Please choose the reason(s) you don't currently own a gun.
Chose to sell or otherwise get rid of the gun(s)
Gun(s) lost or stolen
Some other reason
Don't know

Please choose the reasons you haven't ever owned a gun.
I haven't wanted a gun
I haven't needed a gun
I'm concerned about the safety of children in the house
I'm concerned about risk of suicide or violence
My spouse/partner is uncomfortable with guns
I can't legally have a gun
Some other reason

Does/would having a gun at your home make it a safer place to be, or a more dangerous place to be?
Safer
More dangerous
It depends
Don't know

How often is it appropriate for parents to ask if there are unlocked guns in a home where their children go to play?
Never appropriate
Sometimes appropriate
Usually appropriate
Always appropriate
Don't know

© 2021 Pallin R et al. *JAMA Network Open.*

In general, how often is it appropriate for doctors and other health professionals to talk to their patients about…?*

	Never Appropriate	Sometimes Appropriate	Usually Appropriate	Always Appropriate	Don't know
Seat belt use	☐	☐	☐	☐	☐
Cigarette smoking	☐	☐	☐	☐	☐
Alcohol and drinking	☐	☐	☐	☐	☐
Healthy diet	☐	☐	☐	☐	☐
Physical activity	☐	☐	☐	☐	☐
Gun safety	☐	☐	☐	☐	☐

*The health and safety risks appeared on screen in a random order for each respondent.

In general, how often is it appropriate for doctors or other health professionals to talk to patients about gun safety **when the patient has guns in the home and**…?

	Never Appropriate	Sometimes Appropriate	Usually Appropriate	Always Appropriate	Don't know
is having thoughts of suicide	☐	☐	☐	☐	☐
is having trouble with drugs or alcohol	☐	☐	☐	☐	☐
has dementia or a similar condition	☐	☐	☐	☐	☐
lives with children or teens	☐	☐	☐	☐	☐
lives with someone with dementia or a similar condition	☐	☐	☐	☐	☐

In general, when a doctor learns that their patient has a gun and is thinking of using it to hurt themselves or someone else, how often is it appropriate for the doctor to…?

	Never Appropriate	Sometimes Appropriate	Usually Appropriate	Always Appropriate	Don't know
Counsel the patient not to hurt anyone	☐	☐	☐	☐	☐
Counsel the patient to have someone else keep the gun	☐	☐	☐	☐	☐
Inform the patient's family	☐	☐	☐	☐	☐
Inform the police	☐	☐	☐	☐	☐
Inform a mental health professional	☐	☐	☐	☐	☐

Do you have a political party preference?

The Republican Party
The Libertarian Party
The Democratic Party
The Green Party
The American Independent Party
The Peace and Freedom Party
Other

© 2021 Pallin R et al. *JAMA Network Open.*
